# Comparative analysis of default mode networks in major psychiatric disorders using resting-state EEG

**DOI:** 10.1038/s41598-021-00975-3

**Published:** 2021-11-10

**Authors:** Kang-Min Choi, Jeong-Youn Kim, Yong-Wook Kim, Jung-Won Han, Chang-Hwan Im, Seung-Hwan Lee

**Affiliations:** 1 Clinical Emotion and Cognition Research Laboratory, Inje University, Goyang, Republic of Korea; 2 School of Electronic Engineering, Hanyang University, Seoul, Republic of Korea; 3 Center for Bionics, Korea Institute of Science and Technology (KIST), Seoul, Republic of Korea; 4 Department of Biomedical Engineering, Hanyang University, 222 Wangsimni-ro, Seongdong-gu, Seoul, 04763 Republic of Korea; 5 School of Psychology, Sogang University, Seoul, Republic of Korea; 6 Department of Psychiatry, Ilsan Paik Hospital, Inje University College of Medicine, Juhwa-ro 170, Ilsanseo-Gu, Goyang, 10370 Republic of Korea; 7 Bwave Inc, Juhwa-ro, Goyang, 10380 Republic of Korea

**Keywords:** Electrophysiology, Imaging, Computational models, Computational neuroscience, Psychiatric disorders

## Abstract

Default mode network (DMN) is a set of functional brain structures coherently activated when individuals are in resting-state. In this study, we constructed multi-frequency band resting-state EEG-based DMN functional network models for major psychiatric disorders to easily compare their pathophysiological characteristics. Phase-locking values (PLVs) were evaluated to quantify functional connectivity; global and nodal clustering coefficients (CCs) were evaluated to quantify global and local connectivity patterns of DMN nodes, respectively. DMNs of patients with post-traumatic stress disorder (PTSD), obsessive compulsive disorder (OCD), panic disorder, major depressive disorder (MDD), bipolar disorder, schizophrenia (SZ), mild cognitive impairment (MCI), and Alzheimer’s disease (AD) were constructed relative to their demographically-matched healthy control groups. Overall DMN patterns were then visualized and compared with each other. In global CCs, SZ and AD showed hyper-clustering in the theta band; OCD, MCI, and AD showed hypo-clustering in the low-alpha band; OCD and MDD showed hypo-clustering and hyper-clustering in low-beta, and high-beta bands, respectively. In local CCs, disease-specific patterns were observed. In the PLVs, lowered theta-band functional connectivity between the left lingual gyrus and the left hippocampus was frequently observed. Our comprehensive comparisons suggest EEG-based DMN as a useful vehicle for understanding altered brain networks of major psychiatric disorders.

## Introduction

Default mode network (DMN) is a set of functional brain structures coherently activated when individuals are awake without engaging in any goal-directed activities^1,2^. With the increasing interest in so-called resting-state functional brain activity, studies of the resting-state DMN have been gradually paid attention to for several years^3,4^. Comparison of multiple psychiatric disorders is one of the promising research areas in functional networks. There are several advantages for the study of resting-state functional network of DMN because an individual does not need to be involved in specific tasks requiring attention, which could be easily affected by interrupting factors such as lack of attention due to low motivation or mind wandering^5^.

Relying on numerous advantages, DMN studies have been performed with a variety of modalities such as functional magnetic resonance imaging (fMRI), positron emission tomography (PET), and electroencephalography (EEG)^6–10^. Among them, EEG, which is believed to reflect brain electrical activity directly^11,12^, has recently been regarded as one of the competitive noninvasive and cost-effective modalities for construction of DMN^13,14^. In addition, it can be analyzed into multiple frequency bands, allowing for diverse interpretations according to the characteristics of each frequency band. These beneficial properties help to possess potentials to become employed as a universal evaluation framework of psychiatric disease.

There have been some resting-state EEG-based DMN studies to compare psychiatric disorders and healthy controls (HC). For example, Hsiao et al.^15^ compared resting-state EEG between patients with mild cognitive disorder (MCI) and Alzheimer’s disease (AD) in the DMN, showing various altered interconnections between them. Miraglia et al.^16^ longitudinally compared two types of MCI, one for those who were converted to AD, and the other for those not converted, by the small-worldness index of the DMN. Meanwhile, Krukow et al.^17^ observed theta-band DMN hyperconnectivity in first-episode schizophrenia (SZ) patients, suggesting that it might interfere with efficient cognitive function. Yazdi-Ravandi et al.^18^ also compared multiband EEG-based DMN between patients with obsessive compulsive disorder (OCD) and HC. Likewise, most of the EEG-based DMN studies have focused on the abnormalities of the specific disorder. More recently, a few studies focused on different types of psychiatric disorders. For instance, Zhang et al.^19^ tried to simultaneously identify the subtypes of post-traumatic stress disorder (PTSD) and major depressive disorder (MDD) to predict the treatment effect for them. Cea-Canas et al.^20^ tried to simultaneously compare EEG-based DMN between patients with SZ and bipolar disorder (BD), with the demography of BD not matched with the SZ and HC groups. These simultaneous cross-disorder comparison studies are thought to be more suitable to derive reliable biomarkers from various psychiatric spectra and facilitate association of the analysis results with the neurobiological characteristics, which could broaden the knowledge of the EEG-based DMN. Nevertheless, the studies aiming to compare numerous psychiatric disorders comprehensively are lack until recent times. For example, comparison of DMN between SZ and AD has been actively conducted in the fMRI-based DMN studies owing to their considerable similarity in social deficits^21^; however, it has not been conducted in the EEG-based DMN studies.

In this exploratory study, we tried to construct EEG-based DMN functional network models based on a graph theory for a variety of major psychiatric disorder groups. We wanted to visualize the DMN patterns to readily compare each other, which comprised PTSD, OCD, panic disorder (PD), MDD, BD, SZ, MCI, and AD. We hypothesized that the DMN patterns could easily contrast the characteristics of major psychiatric disorders, and they could reflect the pathophysiology of major psychiatric disorders.

## Methods and materials

### Participants

A database in which patients were diagnosed as having psychiatric disorders from January 2006 to December 2018 from the Inje University Ilsan Paik Hospital was used. A diagnosis of these disorders was based on a clinical evaluation by trained psychiatrists using the Structured Clinical Interview for DSM-IV or V Axis I Disorders (SCID-I) or Mini international neuropsychiatric interview (MINI). Meanwhile, patients who possess neurological or comorbid disorders, other organic brain damage, or impairment in sensory or motor function were excluded from the analysis. Additionally, patients who were in pregnancy were also excluded. Finally, 104 SZ, 74 PTSD, 82 PD, 29 OCD, 69 MDD, 60 BD, 34 MCI, and 29 AD patients were included and analyzed in this study (see Supplementary Table S1 for detailed demographic information and see Supplementary Table S2 for medicine dosage information).

A total of 250 healthy participants were recruited from the local community using advertisements. They satisfied neither the DSM-IV nor V-based lifetime diagnostic criteria for any major psychiatric disorders, as screened by the SCID-I Non-Patient Edition (SCID-NP) nor MINI-based diagnostic criteria. For each disorder group, its corresponding HC participants were selected in pseudorandom for demographic information including age, sex, and education to be matched (Supplementary Table S1).

The ethical approval was made by Inje University Ilsan Paik Hospital Institutional Review Board (IRB no. 2018-12-012-013). The study was carried out in accordance with relevant guidelines and regulations. Because this study was conducted by retrospective data inspection, the informed written consent of patients was waived by the Inje University Ilsan Paik Hospital Institutional Review Board. Data from healthy participants were collected as a study purpose with the written consent (IRB no. 2015-07-025).

### Signal acquisition and pre-processing

Resting-state EEG was recorded for 4 min while the participants closed their eyes in this study. The EEG signal was acquired using the SynAmps amplifier (Neuroscan, Compumedics USA, Charlotte, NC, USA) with 62 Ag/AgCl electrodes mounted on NeuroScan Quik-cap according to the international extended 10–20 system. The additional electrooculogram (EOG) signal was acquired with two electrodes, each attached below the right eye and to the right of the outer canthus. The sampling rate of the equipment was set at 1000 Hz. The recorded signal was band-pass filtered at 0.1–100 Hz and notch-filtered at 60 Hz to remove powerline noise using analogue filter. The impedance of each electrode was maintained below 5 kΩ during the whole experimental period. The ground and reference electrodes were placed on the forehead and both mastoids, respectively.

The acquired EEG data were manually inspected to eliminate segments contaminated by environmental or physiological noises. The eye movement artifact was removed using the mathematical procedure^22^. Subsequently, the signal was applied to common average reference, baseline correction by removing DC offset for each channel, and then segmented into 2 s epochs without an overlap. These series of pre-processing steps were performed using CURRY 7 software (Compumedics NeuroScan; Hamburg, Germany). Among the segmented data, 45 epochs were randomly selected for each participant from the epochs with the maximum absolute value not exceeding 100 μV using MATLAB R2019b (MathWorks; Natick, MA, USA). It is to be noted that no digital filtering was applied in the pre-processing step.

### Construction of the DMN functional network model

#### Regions of interest (ROI)

**Figure 1:**
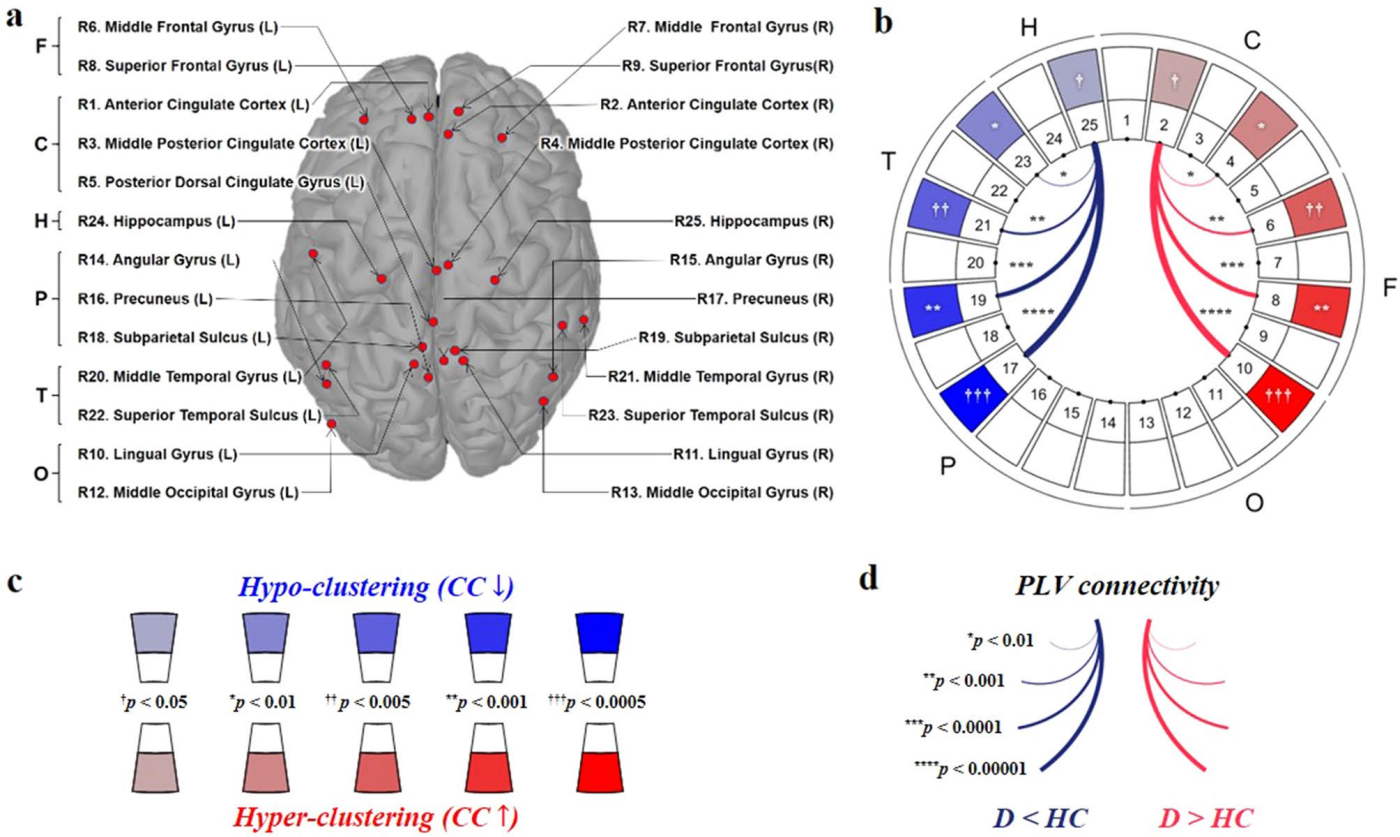
The ROIs of the DMN and an exemplary DMN pattern with significance levels indicated. (**a**) 25 DMN regions are displayed on the brain structure. The presented brain image was acquired from the Brainstorm toolbox. (**b**) The visualized DMN pattern illustrates significantly different local CC index and PLV connectivity of the disorder group compared with its demographically-matched HC group. In the visualization of both properties, red colors indicate the increased properties of the disease groups, while blue colors indicate the opposite case. The external capital letters denote brain regions (C: cingulate, F: frontal, O: occipital, P: parietal, T: temporal, H: hippocampal). (**c**) The significance levels of the CC indices are indicated by the color intensity with five levels. (**d**) The significance level of the PLV connectivity was indicated by the thickness of the curve with the logarithmic scale of the p-value. It is to be noted that only the PLV connectivities, the p-value of which is smaller than 0.01, were illustrated in the pattern. For example in the pattern (**b**), right hippocampus (R25) and right precuneus (R17) showed strong PLV under-connectivity, and right anterior cingulate cortex (R2) showed relatively weak but significant CC hyper-clustering. It is noted that only the connectivities possessing significance levels p < 0.01 were displayed. ^†^p < 0.05, *p < 0.01, ^††^p < 0.005, **p < 0.001, ^†††^p < 0.0005, ***p < 0.0001, ****p < 0.00001.

The determination of the regions of interest (ROIs) and their coordinates was made based on 17 highly cited (> 500) articles that include coordinate information. Among a variety of candidate regions, those referred by more than three times regardless of the hemisphere were selected as the ROIs for DMN. Consequently, 25 DMN ROIs were determined (Fig. 1a), including cingulate (Region 1 ~ 5; R1 ~ 5), frontal (R6 ~ 9), occipital (R10 ~ 13), parietal (R14 ~ 19), and temporal (R20 ~ 23) cortices, and hippocampus (R24 ~ 25). Each coordinate of the ROI was determined as the center of all coordinates for the candidate ROIs.

#### Source localization

To calculate the source activities of ROIs from the recorded scalp EEG signals, a depth-weighted L2-norm estimator implemented in the Brainstorm toolbox^23^ was employed. The Colin27 MRI brain template and the deep brain structures provided by the Brainstorm toolbox were employed to estimate cortical activities and hippocampal activities, respectively. The lead field matrix was constructed using a three-layer boundary element model provided by the OpenMEEG project software^24^. Among the 30,020 nodes each with the estimated cortical current density values, those located within a 5 mm distance from the coordinates of each ROI were selected, and consequently, 25 clusters of nodes corresponding to 25 DMN ROIs were constructed. The source signal of each ROI was then obtained by applying the principal component analysis to the source signals of all nodes in each ROI.

#### Network construction

The DMN functional network model was constructed in accordance with a typical procedure described below: First, the source signals of 25 ROIs (Fig. 1) were evaluated for each epoch as described in the previous paragraph. Second, the source signals of each ROI were decomposed into the following five frequency bands: theta (4–8 Hz), alpha 1 (8–10 Hz), alpha 2 (10–12 Hz), beta 1 (12–18 Hz), and beta 2 (18–30 Hz). This signal decomposition was accomplished by using a 6th order zero-phase Butterworth infinite impulse response (IIR) band-pass filter implemented in the MATLAB Signal Processing toolbox, with the cutoff frequencies equal to the borders of each frequency band. Third, the functional connectivity between every pair of the ROIs was evaluated by the phase-locking value (PLV) that has been widely employed to evaluate phase synchronization^25^. The PLV between each ROI pair was evaluated by averaging PLVs of all 45 epochs for each patient. Fourth, after the functional connectivity network was constructed for each frequency band, the local clustering coefficient (CC) was evaluated to measure the local functional segregation status indicating a degree of nodal clustering within its neighboring nodes^26^. Finally, global CC was evaluated to quantify the overall clustering level of a network.

#### Visualization as DMN pattern

The significance levels of the PLVs and local CCs were expressed by the thickness of the curve between a pair of nodes and the color intensity of the node region (Fig. 1), respectively. In the visualization of both properties, red (or blue) colors in the patterns indicated that the disease group shows significantly higher (or lower) values compared to its corresponding HC group.

### Statistical analysis

All absolute values of skewness were lower than 2, and that of kurtosis was lower than 7 for every demographic distribution, satisfying the normality assumption of those distributions^27^. Two-tailed Student t test was used to examine for age, sex, and education between comparing groups. To avoid multiple test issues, cluster-based permutation test (n = 10,000) was used to test the significance of PLV (n = 12,000) or CC (n = 1040) between pairing groups^28^.

## Results

**Figure 2:**
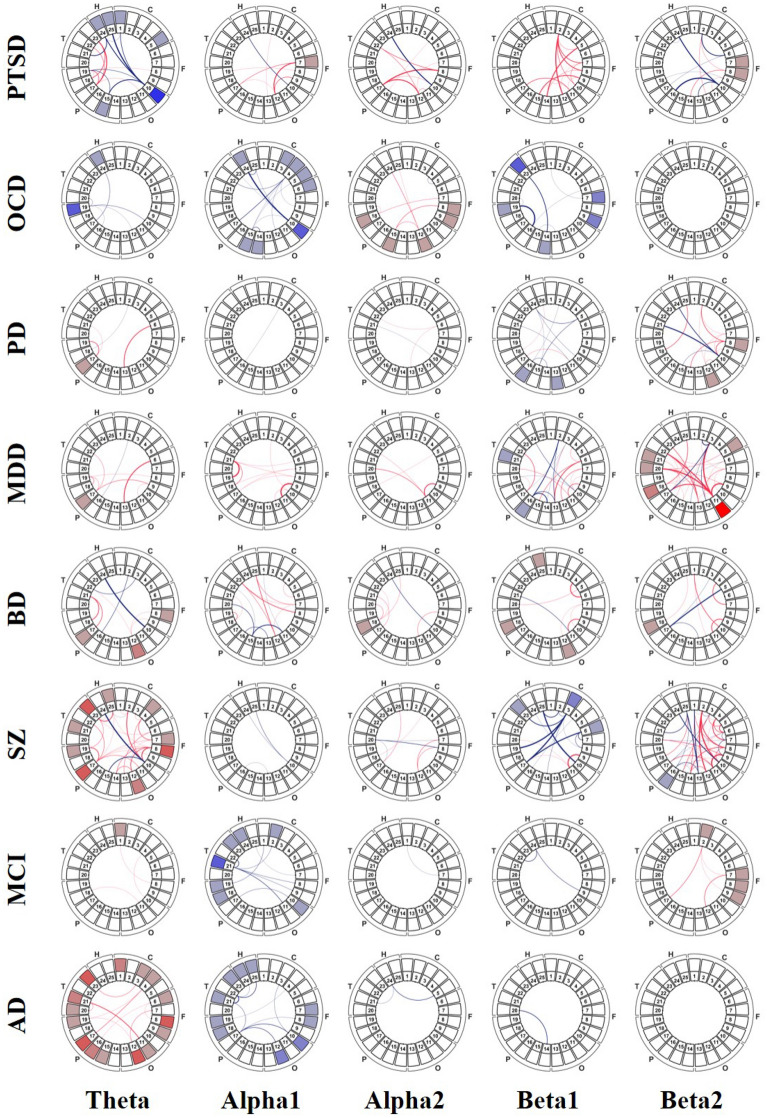
The DMN patterns at a glance in major psychiatric disorders for various frequency bands. The significance levels are illustrated in line with Fig. 1b. (*PTSD* posttraumatic stress disorder, *OCD* obsessive compulsive disorder, *PD* panic disorder, *MDD* major depressive disorder, *BD* bipolar disorder, *SZ* schizophrenia, *MCI* mild cognitive impairment, *AD* Alzheimer’s disease).

**Figure 3:**
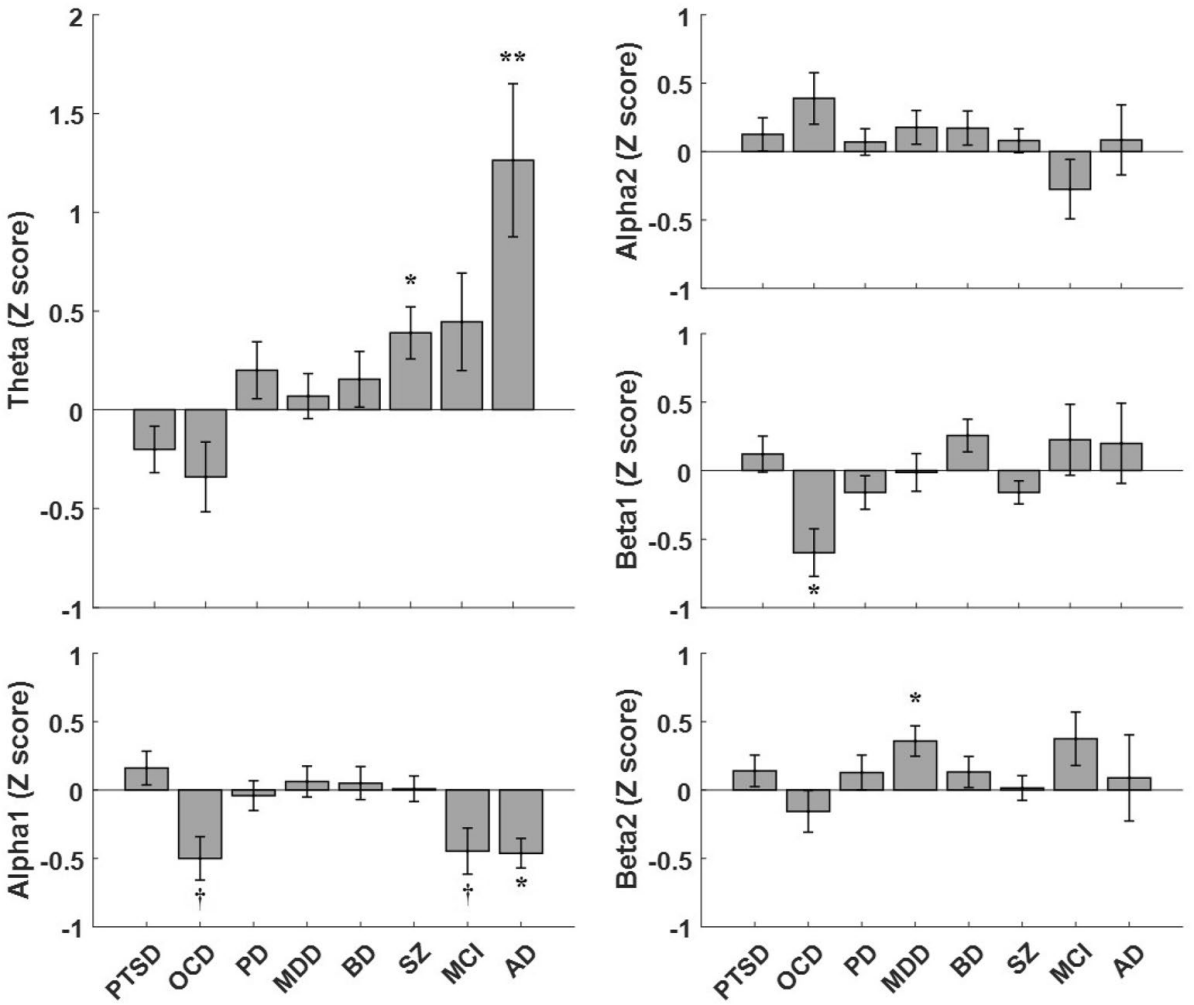
The global CC indices for various frequency bands. The global CC values of each group were normalized to those of the corresponding HC group, using z-transformation. Each bar indicates the averaged z-score of the global CC value of the disorder group. The error-bars indicate standard error. ^†^p < 0.07, *p < 0.05, **p < 0. (*PTSD* posttraumatic stress disorder, *OCD* obsessive compulsive disorder, *PD* panic disorder, *MDD* major depressive disorder, *BD* bipolar disorder, *SZ* schizophrenia, *MCI* mild cognitive impairment, *AD* Alzheimer’s disease).

There were no significant differences between each pair of groups in terms of demographic characteristics, including age, sex, and education (Supplementary Table S1). The functional network model of the DMN was constructed and then visualized based on the determined ROIs (Fig. 1). The significantly different local clustering coefficient (CC) indices and phase-locking value (PLV) connectivity of each disorder group are illustrated at a glance, compared with its demographically matched HC group (Fig. 2). The global CC indices are provided to compare major psychiatric disorders (Fig. 3). DMN patterns of 5 frequency bands were presented based on the frequency bands: theta, alpha1, alpha2, beta1, beta2.

### DMN patterns at a glance

Several differences and similarities were observed in DMN patterns among the disease groups (Fig. 2). First, in CC indices, general homogenous clustering tendencies were found in disease-specific manners. For example, AD, MCI, SZ, and BD showed relative CC hyper-clustering of theta band compared to other disorders. MDD, PD, and PTSD showed relative CC hyper-clustering of beta 2 band compared to other disorders. Second, in PLV connectivity, heterogeneous (mixed) patterns were found in disease-specific manners. For example, SZ and PTSD showed the high and low mixed regional connectivity patterns in theta and beta 2 frequency bands. These mixed connectivity patterns were also observed in beta1 band of MDD.

### Disease-specific patterns of CC indices

Globally, some disease groups showed significantly different global CC indices compared to their corresponding HC groups (Fig. 3; see Supplementary Table S3 for comparison of actual global CC values). In the theta band DMN, significantly higher global CC indices were observed in SZ (p = 0.017) and AD (p = 0.007) groups. In the alpha1 band, significantly lower global CC index was observed in AD (p = 0.036) group; in addition, marginally significantly lower global CC indices were observed in MCI (p = 0.067) and OCD (p = 0.050) groups. In the beta1 band, significantly lower global CC index was observed for OCD (p = 0.022) group; while in the beta2 band, significantly higher global CC index was observed for MCI (p = 0.031) group.

**Figure 4:**
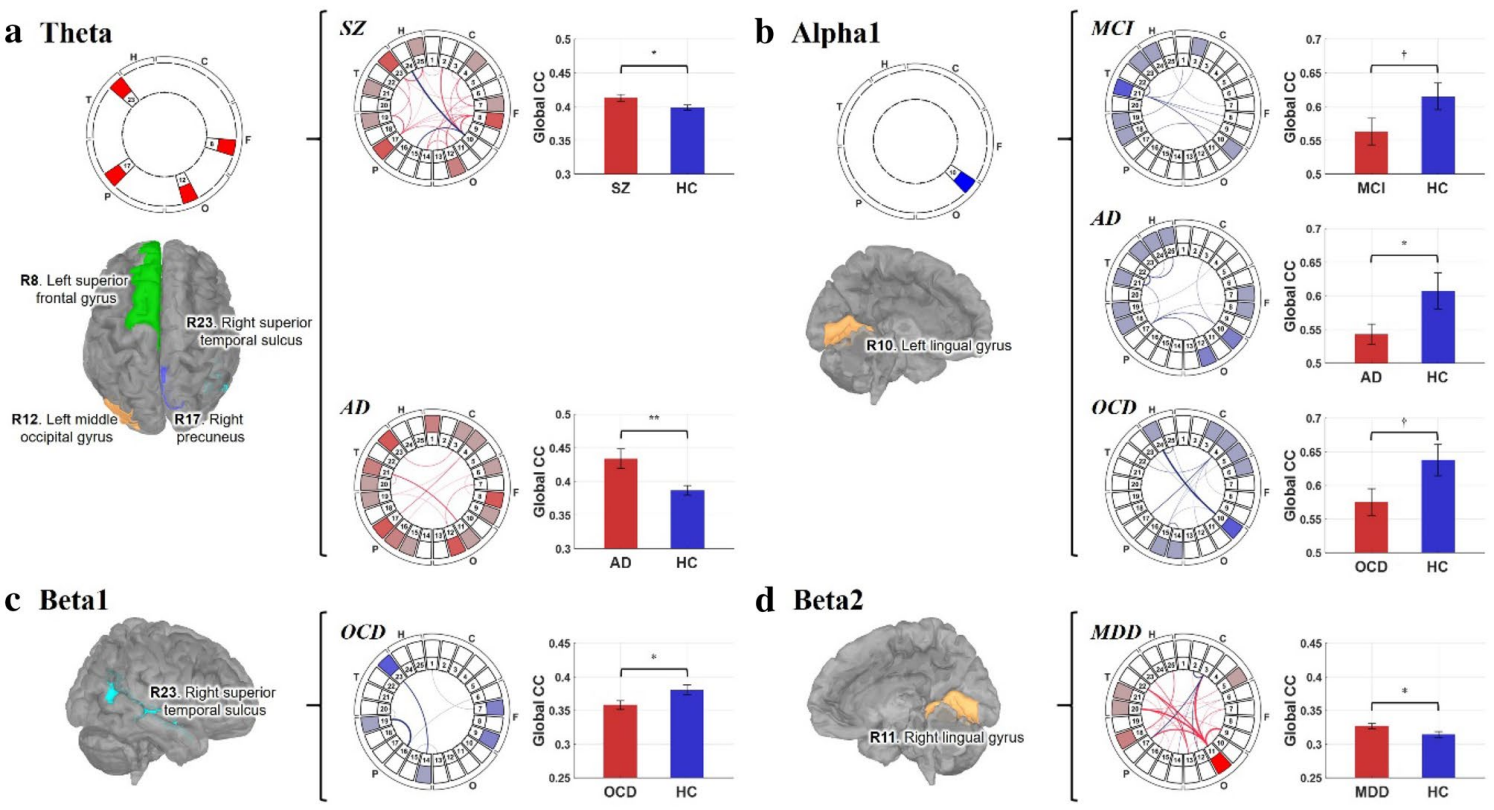
DMN CC index analysis results. Only the results showing significantly different global CC indices are presented. For each frequency band, key ROIs are highlighted on the brain image left side; additionally, if more than one is included, common key ROIs are also illustrated as a form of DMN pattern. DMN patterns and the comparisons of global CC indices are presented right side. In the bar charts, red bars indicate disorder groups, and blue bars show HC groups. The error-bars indicate standard error. (**a**) In the theta band DMN, SZ and AD commonly showed strong hyper-clustering in four regions: left superior frontal gyrus (R8), left middle occipital gyrus (R12), right precuneus (R17), and right superior temporal sulcus (R23). (**b**) In the alpha1 band DMN, MCI, OCD, and AD commonly showed hypo-clustering in the left lingual gyrus (R10). (**c**) In the beta1 band DMN, OCD showed strong hypo-clustering in the right superior temporal sulcus (R23). (**d**) In the beta2 band DMN, MDD showed strong hyper-clustering in the right lingual gyrus (R11). ^†^p < 0.07, *p < 0.05, **p < 0.01 (*SZ* schizophrenia, *AD* Alzheimer’s disease, *OCD* obsessive compulsive disorder, *MCI* mild cognitive impairment, *MDD* major depressive disorder, *HC* healthy control).

Locally, further analyses were performed with some DMN patterns showing aforementioned globally abnormal CC tendencies. In the theta band DMN, SZ and AD groups exhibited similar CC patterns (Fig. 4a): several regions were consistently hyper-clustered in both groups, particularly for left superior frontal gyrus (R8, p = 0.0034 for both groups), left middle occipital gyrus (R12, p = 0.0072 for SZ; p = 0.0013 for AD), right precuneus (R17, p = 0.0014 for SZ; p = 0.0045 for AD), and right superior temporal sulcus (R23, p = 0.0024 for SZ; p = 0.0019 for AD). In the alpha1 band, OCD, MCI, and AD groups exhibited widespread lower local CC patterns (Fig. 4b). Although each of them exhibited a common CC hypo-clustering in left lingual gyrus (R10, p = 0.0449 for MCI; p = 0.0100 for AD; p = 0.0033 for OCD), the DMN patterns were quite different from each other. For Example, the OCD group showed significantly lower CC indices mainly in the posterior cingulate cortex (R3 ~ 5), whereas MCI and AD groups showed little difference in the regions. Meanwhile, the AD group showed widespread hypo-clustered regions compared to the MCI group. In the beta1 band, the OCD group showed strong CC hypo-clustering in the right superior temporal sulcus (R23, p = 0.0030; Fig. 4c). In the beta2 band, the MDD group showed strong CC hyper-clustering in the right lingual gyrus (R11, p = 0.0013; Fig. 4d). These high frequency band DMN patterns exhibited focal abnormal CC characteristics.

### Disease-specific patterns of PLV connectivity

**Figure 5:**
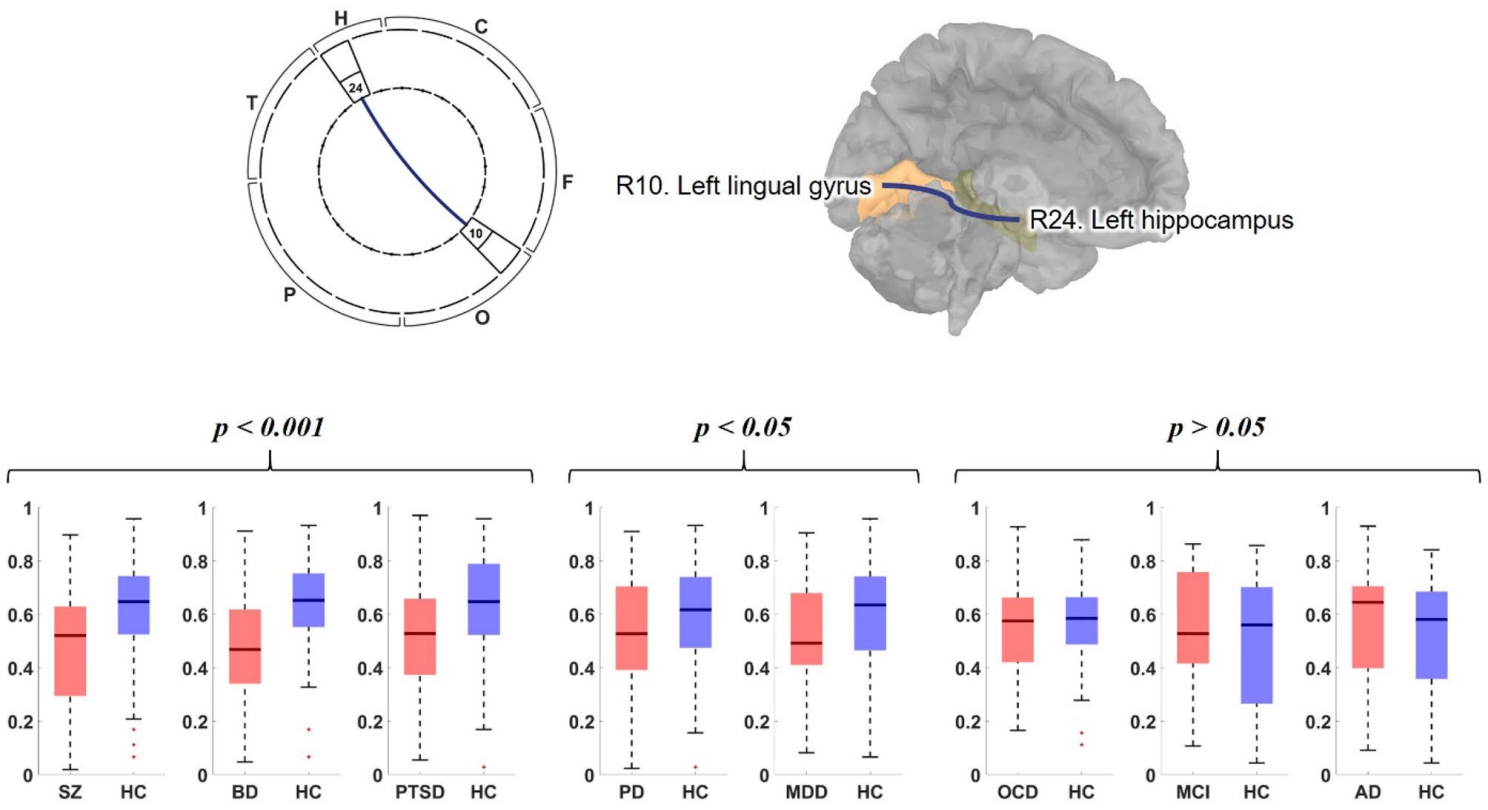
Comparison of theta band PLV connectivity between left lingual gyrus (R10) and left hippocampus (R24) for all disorder groups (red boxes) with their corresponding HC groups (blue boxes). The connectivity is illustrated as a form of DMN pattern (top left), which is highlighted on the brain image (top right). In the box plots (bottom), the black midlines indicate median values, the boxes indicate interquartile range (IQR), whiskers indicate the maximum and minimum value in the 1.5 times of IQR, and crosses (+) indicate outliers. The comparison results are displayed with three classes: the first class (SZ, BD, and PTSD) showed strongly decreased connectivity (p < 0.001); the second class (PD and MDD) showed relatively weak but significantly decreased connectivity (p < 0.05); and the third class (OCD, MCI, and AD) showed no significantly different connectivity. (*SZ* schizophrenia, *BD* bipolar disorder, *PTSD* posttraumatic stress disorder, *PD* panic disorder, *MDD* major depressive disorder, *OCD* obsessive compulsive disorder, *MCI* mild cognitive impairment, *AD* Alzheimer’s disease, *HC* healthy control).

Interestingly, lowered functional connectivity of theta band between left lingual gyrus (R10: region number 10 in Fig. 1a,b) and left hippocampus (R24: region number 24 in Fig. 1a,b) was frequently observed over all disease (Fig. 5). However, statistically significant differences were found in the PTSD, BD, and SZ groups (p < 0.001 for BD and PTSD groups; p < 0.0001 for SZ group), and in the PD and MDD (p = 0.016, and p = 0.030, respectively).

## Discussion

In the present study, EEG-based DMN functional network models were constructed for eight major psychiatric disorders based on a graph theory. The constructed DMNs were visualized, and then their DMN patterns were compared with each other. Overall DMN patterns were well visualized at a glance. Abnormal DMN patterns can be discussed in three types of properties, compared to the values of HCs. First, global hyper (hypo)-clustering reflects overall higher (lower) functional connectivity tendencies within the network. Second, distinctive local hyper (hypo)-clustering compared to other regions might reflect abnormally higher (lower) activation or function centering around the specific region of the brain. Third, higher (lower) PLV connectivity between a couple of regions reflects increased (decreased) functional connectivity itself between them. Hence, the disease-specific global DMN patterns were discussed first and then several key local CC and PLV patterns were discussed.

### DMN patterns at a glance

DMN CC indices exhibited homogeneous alteration patterns according to their specific symptom. Disease groups known to show cognitive decline such as AD, MCI, SZ, and BD^17,29^ exhibited relatively hyper-clustering patterns of theta band compared to the others (discussed in the following section). On the other hand, disease groups known to show anxiety symptoms exhibited relatively hyper-clustering patterns of high frequency bands compared to the others. For example, PTSD, PD, and MDD showed relatively higher CC indices of beta2 frequency band; OCD showed relatively higher CC indices of alpha2 frequency band. These symptom-dependent alterations of the local CC tendencies in a specific frequency band are thought to be associated with particular pathophysiological symptoms (more detail in the following section).

Most PLV connectivity showed mixed patterns, which may imply distinct region-specific activation patterns, even in single frequency bands in a specific disease. These mixed patterns were dominantly observed in MDD and SZ. MDD and SZ are very heterogeneous disease entities among psychiatric disorders^30,31^. Even in single diagnostic entity, each patient could show a distinct clustering pattern in DMN brain regions^31^. Our results agree with the previous studies reporting that MDD and SZ exhibited abnormal mixed functional patterns^32,33^.

### Disease-specific patterns of CC indices

In the theta band CC, the SZ and AD groups showed predominant global hyper-clustering. Our results agree with the previous reports in which dominantly hyper-clustered theta band DMN might reflect inefficient cognitive function^34^. Additional previous studies reported that whole-brain theta band functional connectivity increased in the patients with SZ and AD^34,35^. In our study, both SZ and AD showed particularly higher CC indices for some common ROIs, including the left superior frontal gyrus (R8), left middle occipital gyrus (R12), right precuneus (R17), and right superior temporal gyrus (R23). The theta band hyper-clustering of fronto-temporo-patietal regions has been consistently reported in SZ and AD^34,35^, which is linked to working and verbal memory. Theta hyper-clustering of the occipital cortex might involve psychiatric symptoms such as dysfunction (i.e. illusion or hallucination) of the visual system^36^, which is commonly observed in both disorders. However, the AD group showed more widespread hyper-clustering than the SZ group, implying more severe neuronal degeneration.

In the alpha1 band CC, the OCD, MCI, and AD groups showed consistent global hypo-clustering. In the previous studies, interregional alpha band synchrony is thought to be related to the coordinating functional integration^37^. The deterioration of functional integration can easily affect the cognitive decline. Therefore, our findings support that cognitive function decline is one of the consistent symptoms not only in patients with MCI and AD but also in patients with OCD^35,38^. Considering this significant deterioration was not observed in the alpha2 band, the integrational function might be more related to the low alpha (alpha 1) band synchrony. Locally, our study revealed that the left lingual gyrus (R10) was commonly hypo-clustered in all groups. The lingual gyrus is linked to the visual system^39,40^. Meanwhile, in the MCI group, the hypo-clustered regions include mainly the parietotemporal cortex, especially for the right middle temporal cortex (R21). However, in the AD group, the regions were more spread broadly including the fronto-occipital cortex and hippocampus. The regional broadening involved suggests a progression of neuronal degeneration from MCI to AD. Generally, it is well known that structural or functional abnormality begins at the temporal lobe in the patients with early state MCI, and then gradually broadened to other brain regions in patients with AD^39^. In the OCD group, the main hypo-clustered region was the posterior cingulate cortex, distinctively from other disease groups. Previous works reported that the gray matter volume and resting-state metabolism of the posterior cingulate cortex consistently increased in OCD patients^41,42^, different from the other groups. Consequently, it can be presumed that resting-state functional hypo-clustering of the posterior cingulate cortex in the OCD group is actually caused by excessive mental load such as obsessive rumination.

In the beta1 band CC, the OCD group showed predominant global hypo-clustering, particularly in the right superior temporal sulcus. Meanwhile, in the beta2 band CC, the MDD group showed predominant global hyperactivation, particularly in the right lingual gyrus. These region-specific differences were also reported in previous brain volumetric studies. The cortical volume of the right superior temporal gyrus (R23) was decreased in the patients with OCD^40^, and that of the right lingual gyrus (R11) was increased in the patients with MDD^43^. Our results showing the differences in local CC index might be attributed to the brain morphological differences.

### Disease-specific patterns of PLV connectivity

The lowered functional connectivity of theta band between the left lingual gyrus (R10) and the left hippocampus (R24) in the theta band DMN was predominantly observed in the BD, SZ, PTSD, PD, and MDD groups. Hippocampal theta rhythm is associated with episodic memory^44^, and lingual gyrus is linked to encoding and retrieval of the visual memory^39,45^. Meanwhile, cognitive decline is well known pathophysiology of those psychiatric disorders, including PTSD^39,46–52^. Although a cognitive decline of PTSD could be a somewhat controversial issue, there are many studies documenting it, ranging from young adults^46^ to the elderly with PTSD^47,48^. Furthermore, a cognitive decline of PTSD might also be inferred from a study reporting that patients with PTSD are more vulnerable to future dementia^53^. Thus, our results might be related to the decline of cognitive function.

### Effects of the psychotropic medication

The effects of the psychotropic medication on a cognitive functioning and EEG have not been reached to the consensus, due to the highly heterogeneous results or lack of studies^54–58^. For example, the relationship between cognition and antipsychotics medication still remains controversial: some studies reported the beneficial effect, but other studies even reported the adverse effects on cognition^54^. The effects on the band power of EEG were not totally reached to the consensus^59^: not only the inter-drug studies for the same type such as lithium, carbamazepine, lamotrigine, and valproate, all of which are included in the mood stabilizer; but intra-drug studies, such as haloperidol, a type of antipsychotics agent. Although some studies reported no significant effects^20^, the effects on the functional connectivity show heterogeneous results, which rendered it difficult to reach the consensus^60^. To sum up, the effects of psychotropic medication on cognitive functioning and EEG are known to be very difficult to be specified due to the heterogeneous results. Further studies are required to control the quantitative effect of the drugs to provide more reliable experimental results.

There are some limitations in our study. First, some disease groups have relatively small numbers of participants. Second, the patients were not obtained in the drug naive state. Third, although assuming that some abnormal DMN characteristics might be related to a cognitive function, we do not have a cognitive assessment data underpinning this argument. Hence, future studies are required to reveal the relationship between these findings and cognitive function. Finally, symptomatic severity was not controlled.

In conclusion, we tried to compare the resting-state multiband EEG-based DMN for major psychiatric disorder groups by patterning it to be visualized at a glance. As expected, a variety of disease-specific DMN patterns were observed, which might be linked to the neurobiological characteristics. Our results showed that EEG-based DMN is a clinically useful and pathologically relevant method to evaluate major psychiatric disorders. Future work is needed to explore the relationship between DMN and symptomatic severity.

## Supplementary Information

Supplementary Tables.
