# Author Correction: Comparative analysis of default mode networks in major psychiatric disorders using resting-state EEG

**DOI:** 10.1038/s41598-021-03736-4

**Published:** 2021-12-14

**Authors:** Kang‑Min Choi, Jeong‑Youn Kim, Yong‑Wook Kim, Jung‑Won Han, Chang‑Hwan Im, Seung‑Hwan Lee

**Affiliations:** 1 Clinical Emotion and Cognition Research Laboratory, Inje University, Goyang, Republic of Korea; 2 School of Electronic Engineering, Hanyang University, Seoul, Republic of Korea; 3 Center for Bionics, Korea Institute of Science and Technology (KIST), Seoul, Republic of Korea; 4 Department of Biomedical Engineering, Hanyang University, 222 Wangsimni‑ro, Seongdong‑gu, Seoul, 04763 Republic of Korea; 5 School of Psychology, Sogang University, Seoul, Republic of Korea; 6 Department of Psychiatry, Ilsan Paik Hospital, Inje University College of Medicine, Juhwa‑ro 170, Ilsanseo‑Gu, Goyang, 10370 Republic of Korea; 7 Bwave Inc, Juhwa-ro, Goyang, 10380 Republic of Korea

Correction to: *Scientific Reports* 10.1038/s41598-021-00975-3, published online 10 November 2021

The original version of this Article contained an error in the Acknowledgments section.

“This work was supported by the Brain Research Program through the National Research Foundation of Korea from the Ministry of Science, ICT & Future Planning (NRF-2015M3C7A1028252) and the Korea Medical Device Development Fund grant funded by the Korea government (the Ministry of Science and ICT, the Ministry of Trade, Industry and Energy, the Ministry of Health & Welfare, Republic of Korea, the Ministry of Food and Drug Safety) (Project Number: 202013B10) to Seung Hwan Lee.”

now reads:

"This work was supported by the Korea Medical Device Development Fund grant funded by the Korea government (the Ministry of Science and ICT, the Ministry of Trade, Industry and Energy, the Ministry of Health &Welfare, the Ministry of Food and Drug Safety) (1711138348, KMDF_PR_20200901_0169)."

The original Article has been corrected.

