# Supplementary material for: Comparative analysis of default mode networks in major psychiatric disorders using resting-state EEG
Source: Sci Rep. 2021 Nov 10;11:22007. doi: 10.1038/s41598-021-00975-3 (PMC8580995; doi:10.1038/s41598-021-00975-3)
Supplement: Supplementary file 1 — Supplementary Tables. [file 41598_2021_975_MOESM1_ESM.docx]

**Supplementary Materials**

**Supplementary Table 1**. The demographic information for all psychiatric disorder groups with their corresponding healthy control (HC) groups. In each disorder group, ‘D’ denotes the disorder group, and ‘H’ denotes its corresponding HC group.

|  | **SZ** | | **PTSD** | | | | **PD** | | | | **OCD** | | | | **MDD** | | | | **BD** | | | | **MCI** | | | | **AD** | | | |
| --- | --- | --- | --- | --- | --- | --- | --- | --- | --- | --- | --- | --- | --- | --- | --- | --- | --- | --- | --- | --- | --- | --- | --- | --- | --- | --- | --- | --- | --- | --- |
|  | D | H | D | | H | | D | | H | | D | | H | | D | | H | | D | | H | | D | | H | | D | | H | |
| **N** | 104 | 104 | 74 | 74 | | 82 | | 81 | | 29 | | 29 | | 69 | | 69 | | 60 | | 59 | | 34 | | 35 | | 29 | | 27 | |  |
| **Age** |  |  |  |  | |  | |  | |  | |  | |  | |  | |  | |  | |  | |  | |  | |  | |  |
| Mean  (SD) | 36.35  (12.75) | 38.04  (12.21) | 44.16  (13.48) | 45.14  (13.5) | | 39.9  (9.83) | | 41.53  (10.27) | | 27.76  (9.51) | | 30.55  (9.70) | | 42.33  (10.63) | | 43.41  (11.02) | | 34.25  (10.42) | | 36.19  (10.69) | | 74.59  (7.01) | | 71.74  (5.19) | | 76.24  (5.09) | | 73.78  (4.15) | |  |
| *p* | 0.329 | | 0.661 | | | 0.303 | | | | 0.273 | | | | 0.562 | | | | 0.319 | | | | 0.061 | | | | 0.053 | | | |  |
| **Sex** |  |  |  |  | |  | |  | |  | |  | |  | |  | |  | |  | |  | |  | |  | |  | |  |
| M (%) | 45  (43.3) | 39  (37.5) | 28  (37.8) | 22  (29.7) | | 41  (50.0) | | 32  (39.5) | | 15  (51.7) | | 14  (48.3) | | 26  (37.7) | | 23  (33.3) | | 25  (41.7) | | 21  (35.6) | | 13  (38.2) | | 11  (31.4) | | 5  (17.2) | | 5  (18.5) | |  |
| F (%) | 59  (56.7) | 65  (62.5) | 46  (62.2) | 52  (70.3) | | 41  (50.0) | | 49  (60.5) | | 14  (48.3) | | 15  (51.7) | | 43  (62.3) | | 46  (66.7) | | 35  (58.3) | | 38  (64.4) | | 21  (61.8) | | 14  (68.6) | | 24  (82.8) | | 22  (81.5) | |  |
| *p* | 0.397 | | 0.297 | | | 0.178 | | | | 0.793 | | | | 0.594 | | | | 0.496 | | | | 0.442 | | | | 0.901 | | | |  |
| **Education** |  |  |  |  | |  | |  | |  | |  | |  | |  | |  | |  | |  | |  | |  | |  | |  |
| Mean  (SD) | 13.1  (2.71) | 13.22  (2.52) | 12.77  (2.98) | 13.32  (3.79) | | 13.85  (2.23) | | 14.29  (3.03) | | 13.37  (2.13) | | 14.59  (2.64) | | 13.52  (3.32) | | 14.03  (2.72) | | 12.82  (3.52) | | 12.64  (2.21) | | 8.32  (4.7) | | 10.29  (5.24) | | 5.66  (4.03) | | 7.96  (5.38) | |  |
| *p* | 0.754 | | 0.333 | | | 0.309 | | | | 0.064 | | | | 0.33 | | | | 0.741 | | | | 0.107 | | | | 0.077 | | | |  |

(SZ: schizophrenia, PTSD: posttraumatic stress disorder, PD: panic disorder, OCD: obsessive compulsive disorder, MDD: major depressive disorder, BD: bipolar disorder, MCI: mild cognitive impairment, AD: Alzheimer’s disease)

**Supplementary Table 2.** The medication dosage information for all psychiatric disorder groups. The mean doses are presented with the standard deviation being in the parentheses. The doses of antipsychotics, benzodiazepine, mood stabilizer, and antidepressants were equivalent (mg) to the chlorpromazine, diazepam, valproate, and fluoxetine, respectively.

|  | **SZ** | **PTSD** | **PD** | **OCD** | **MDD** | **BD** | **MCI** | **AD** |
| --- | --- | --- | --- | --- | --- | --- | --- | --- |
| **Antipsychotics** | 1340.76  (3240.94) | 53.25  (102.64) | 2.14  (13.77) | - | 18.53  (99.59) | 300.30  (394.49) | - | 23.04  (67.62) |
| **Benzodiazepine** | 6.35  (9.12) | 8.40  (10.82) | 2.30  (5.14) | 1.87  (4.89) | 2.77  (5.96) | 4.50  (7.57) | 0.05  (0.19) | 0.66  (1.96) |
| **Mood stabilizer** | 162.04  (456.87) | 55.32  (217.53) | - | - | 55.25  (436.65) | 548.23  (609.67) | - | - |
| **Antidepressants** | 2.62  (7.79) | 17.87  (20.82) | 8.15  (9.67) | 9.90  (17.06) | 8.48  (11.22) | 3.51  (12.50) | 0.76  (3.12) | 4.95  (9.14) |

(SZ: schizophrenia, PTSD: posttraumatic stress disorder, PD: panic disorder, OCD: obsessive compulsive disorder, MDD: major depressive disorder, BD: bipolar disorder, MCI: mild cognitive impairment, AD: Alzheimer’s disease)

**Supplementary Table 3.** The global clustering coefficient (CC) information for all psychiatric disorder groups with their corresponding HC groups. In each disorder group, ‘D’ denotes the disorder group, and ‘H’ denotes its corresponding HC group.

|  | SZ | | PTSD | | PD | | OCD | | MDD | | BD | | MCI | | AD | |
| --- | --- | --- | --- | --- | --- | --- | --- | --- | --- | --- | --- | --- | --- | --- | --- | --- |
|  | D | H | D | H | D | H | D | H | D | H | D | H | D | H | D | H |
| **Theta** |  |  |  |  |  |  |  |  |  |  |  |  |  |  |  |  |
| CC | 0.413  (0.050) | 0.399  (0.037) | 0.390  (0.041) | 0.398  (0.041) | 0.402  (0.050) | 0.394  (0.038) | 0.388  (0.042) | 0.403  (0.044) | 0.400  (0.038) | 0.397  (0.04) | 0.398  (0.035) | 0.393  (0.032) | 0.412  (0.072) | 0.390  (0.050) | 0.434  (0.078) | 0.387  (0.037) |
| *p* | **0.0167^*^** | | 0.2263 | | 0.2815 | | 0.1964 | | 0.6801 | | 0.4356 | | 0.1405 | | **0.007^**^** | |
| **Alpha1** |  |  |  |  |  |  |  |  |  |  |  |  |  |  |  |  |
| CC | 0.598  (0.116) | 0.597  (0.122) | 0.601  (0.118) | 0.583  (0.112) | 0.593  (0.123) | 0.598  (0.125) | 0.575  (0.107) | 0.638  (0.125) | 0.612  (0.125) | 0.604  (0.133) | 0.583  (0.101) | 0.578  (0.109) | 0.563  (0.115) | 0.615  (0.117) | 0.543  (0.081) | 0.607  (0.139) |
| *p* | 0.9522 | | 0.3441 | | 0.7843 | | **0.0502^†^** | | 0.7126 | | 0.7895 | | **0.0673^†^** | | **0.0365^*^** | |
| **Alpha2** |  |  |  |  |  |  |  |  |  |  |  |  |  |  |  |  |
| CC | 0.563  (0.088) | 0.555  (0.099) | 0.560  (0.106) | 0.548  (0.101) | 0.564  (0.096) | 0.556  (0.109) | 0.595  (0.098) | 0.557  (0.097) | 0.578  (0.105) | 0.560  (0.102) | 0.570  (0.102) | 0.552  (0.106) | 0.525  (0.088) | 0.544  (0.070) | 0.538  (0.091) | 0.533  (0.066) |
| *p* | 0.5422 | | 0.4616 | | 0.6401 | | 0.1504 | | 0.3093 | | 0.3449 | | 0.3243 | | 0.8018 | |
| **Beta1** |  |  |  |  |  |  |  |  |  |  |  |  |  |  |  |  |
| CC | 0.371  (0.043) | 0.379  (0.049) | 0.368  (0.044) | 0.363  (0.038) | 0.366  (0.050) | 0.373  (0.045) | 0.358  (0.035) | 0.381  (0.038) | 0.371  (0.040) | 0.371  (0.035) | 0.383  (0.040) | 0.371  (0.043) | 0.367  (0.037) | 0.361  (0.025) | 0.371  (0.055) | 0.364  (0.034) |
| *p* | 0.2163 | | 0.5089 | | 0.3340 | | **0.0220^*^** | | 0.9406 | | 0.1530 | | 0.4727 | | 0.5804 | |
| **Beta2** |  |  |  |  |  |  |  |  |  |  |  |  |  |  |  |  |
| CC | 0.323  (0.039) | 0.323  (0.042) | 0.320  (0.029) | 0.316  (0.030) | 0.323  (0.038) | 0.319  (0.033) | 0.319  (0.039) | 0.327  (0.048) | 0.327  (0.032) | 0.314  (0.035) | 0.327  (0.032) | 0.322  (0.036) | 0.335  (0.035) | 0.324  (0.03) | 0.321  (0.037) | 0.319  (0.022) |
| *p* | 0.9200 | | 0.4025 | | 0.4568 | | 0.5309 | | **0.0311^*^** | | 0.4514 | | 0.1515 | | 0.8149 | |

† p < 0.07, * p < 0.05, ** p < 0.01

(SZ: schizophrenia, PTSD: posttraumatic stress disorder, PD: panic disorder, OCD: obsessive compulsive disorder, MDD: major depressive disorder, BD: bipolar disorder, MCI: mild cognitive impairment, AD: Alzheimer’s disease)
